# 

**Published:** 1859-01

**Authors:** 


NORTH AMERICAN

### MEDICO-CHIRURGICAL REVIEW.

Vol. III.] JANUARY, 1859. [No. 1.

### CONTENTS.

### ANALYTICAL AND CRITICAL REVIEWS.

ART. PAGE

I.—On the Diseases, Injuries, and Malformations of the Rectum and Anus. By T. J. Ashton, M.D. . . . . . . . 1

II.—Proceedings of the Ophthalmological Congress of Brussels. By Dr. Warlomont, Secretary-General, etc. . . . . . 22

III.—Epilepsy and Other Convulsive Affections; their Pathology and Treatment. By Charles Bland Radcliffe, M.D. . . . 34

IV.—A Treatise on Fractures. By J. F. Malgaigne, M.D. Translated from the French, by John H. Packard, M.D. . . . 50

V.—Diseases of the Urinary Organs: a Compendium of their Diagnosis, Pathology, and Treatment. By William Wallace Morland, M.D. . . . . . . . . . . . . . 55

VI.—The Ophthalmoscope; its Mode of Application Explained, etc. By Jabez Hogg, M.D. . . . . . . . . . 60

VII.—A System of Human Anatomy, General and Special. By Erasmus Wilson, F.R.S. . . . . . . . . . . . 63

VIII.—Lectures on the Principles and Practice of Physic. By Thomas Watson, M.D. . . . . . . . . . . 63

### ORIGINAL COMMUNICATIONS.

I.—Contributions to Obstetric Jurisprudence. By Horatio R. Storer, M.D. . . . . . . . . . . . . . . . 64

II.—A Case of Aneurism of the Right Femoral Artery Cured by Digital Compression. By Samuel W. Gross, M.D. . . . . 73

III.—Case of Congenital Cataract; Total Blindness for Sixteen Years; Operation, and Subsequent Enjoyment of Sight. By John S. Rohrer, M.D. . . . . . . . . . . . 85

IV.—A Case of Uterine Hemorrhage Three Weeks after Delivery. By G. A. Kunkler, M.D. . . . . . . . . . . . 89

V.—Proceedings of the Pathological Society of Philadelphia. . . 91

### AMERICAN PROGRESS OF MEDICAL SCIENCE.

Report on the Diseases of Children. By D. Francis Condie, M.D. . . . 120

### BI-MONTHLY FOREIGN ABSTRACTS.

PATHOLOGY.

I.—On Ulceration and Perforation of the Diaphragm in Peritonitis. By Dr. E. Bonamy . . . . . . . . . . 146

ART. SURGERY. PAGE

II.—On Polypus of the Œsophagus. By Prof. A. H. MIDDELDORPFF. 147
III.—On Compound Luxations. By Dr. ALBERT SCHINZINGER . . . 149

OBSTETRICS.

IV.—On the Puerperal Diseases Observed in the Charité of Berlin. By Prof. VIRCHOW. . . . . . . . . . . 152

BI-MONTHLY PERISCOPE.

SURGERY.

1. On the Theory of the Production of Hernia . . . . . . 154
2. Syphilization; its Mode of Performance and its Results . . . 156
3. Excision of the Tongue for Cancer . . . . . . . . 163
4. Displacement of the Testicle into the Perinæum; Plastic Operation Unsuccessful; Castration . . . . . . . . . . 167

PRACTICE OF MEDICINE.

1. Practical Observations on Cardialgia . . . . . . . . 168
2. On the Treatment of Croup . . . . . . . . . . . 170
3. On the Introduction of a Tube into the Larynx in Croup . . . 174

OBSTETRICS.

1. On Aloes Enemata in Uterine Catarrh . . . . . . . . . 175
2. On the Prevention of Laceration of the Perinæum . . . . . 177
3. Eclampsia at the Eighth Month; Recovery; Delivery of Two Children United by their Sides . . . . . . . . . . . . 178

MATERIA MEDICA AND THERAPEUTICS.

1. Tartar Emetic in Large Doses in the Treatment of Chorea . . . . 179
2. On the Use of Propylamin in Rheumatism . . . . . . . . 180
3. On the External Use of Hydrochloric Acid . . . . . . 180
4. Ergotin in Pulmonary Tuberculosis . . . . . . . . . 181
5. Acetate of Copper in Pruritis . . . . . . . . . . 181
6. Nitrate of Silver in the Treatment of Ozæna . . . . . . 181
7. Researches on the Therapeutical Action of the Perchloride of Iron in the Treatment of Acute and Chronic Urethritis. . . . . . 181

EDITORS' TABLE.—Geological Affinities of Thermal Springs . . . . 183
"A Hit—A Very Palpable Hit" . . . . . . . . . . 187
Retention of an Enormous Quantity of Urine . . . . . . . 188
A New Preparation of Cimicifuga Racemosa . . . . . . . 189
Blockley Hospital . . . . . . . . . . . . . . 189
Criminal Abortion . . . . . . . . . . . . . . 189
Quackery in Paris . . . . . . . . . . . . . . 189
M. Falcony's Powder for Preserving Dead Bodies. . . . . . 190
Treatment of the Apparently Drowned . . . . . . . . 190

BI-MONTHLY BIBLIOGRAPHICAL RECORD . . . . . . . . . . 192

